# Factors associated with clinical outcome in 25 patients with avian influenza A (H7N9) infection in Guangzhou, China

**DOI:** 10.1186/s12879-016-1840-4

**Published:** 2016-10-03

**Authors:** Hui Wang, XinCai Xiao, Jianyun Lu, Zongqiu Chen, Kuibiao Li, Hui Liu, Lei Luo, Ming Wang, ZhiCong Yang

**Affiliations:** Guangzhou Centre for Disease Control and Prevention, No. 1, Qide Rd, Jiahe, Baiyun, Guangzhou, 510440 China

**Keywords:** Avian influenza, H7N9 subtype, Clinical characteristic

## Abstract

**Background:**

Guangzhou reported its first laboratory-confirmed case of influenza A (H7N9) on January 10, 2014. A total of 25 cases were reported from the first wave of the epidemic until April 8, 2014. The fatality rate was much higher than in previous reports. The objective of the current work was to describe the clinical and epidemiological characteristics of A (H7N9) patients in Guangzhou and explore possible reasons for the high fatality rate.

**Methods:**

Clinical and epidemiological information regarding A (H7N9) cases in Guangzhou was collected through review of medical records and field research. Data regarding clinical and laboratory features, treatment, and outcomes were extracted.

**Results:**

Of the 25 patients, 84 % (21/25) had one or more underlying diseases. Fifteen patients (60.0 %) developed moderate to severe acute respiratory distress syndrome (ARDS), and 14 (56 %) died of the ARDS or multiorgan failure. Patients with longer delay between onset of illness and initiation of oseltamivir treatment were more likely to develop ARDS. Elevated C-creative protein, aspartate aminotransferase, creatine kinase, and lymphocytopenia predicted a higher risk of developing ARDS.

**Conclusions:**

The presence of underlying diseases and clinical complications predicted poor clinical outcome. Early oseltamivir treatment was associated with a reduced risk of developing ARDS.

## Background

The emergence of a novel avian-origin influenza virus subtype from patients with severe and fatal respiratory disease has received global attention since the first reported case of human infection with avian influenza A (H7N9) virus (A (H7N9)) on March 31, 2013. As of 8 April 2014, China has experienced two epidemic waves, and 402 A (H7N9) laboratory-confirmed cases and 146 deaths have been reported [1].

Patients infected with A (H7N9) virus often experience rapidly progressing pneumonia, respiratory failure, and acute respiratory distress syndrome(ARDS), which can be fatal. As previously reported, patients with A (H7N9) infection always show systemic organ injury involving the circulation, kidneys, or liver [2, 3]. Several studies have suggested that the clinical outcomes for A (H7N9) patients correlate with severity of their symptoms, laboratory data, or important medical timelines. For example, severely affected patients showed a significantly longer interval from the onset of illness to antiviral treatment and to the negative conversion of A (H7N9) [4]. Patients who died tended to display elevated average levels of alanine aminotransferase (ALT) and aspartate aminotransferase (AST) [5]. Chen and Shen have also reported that higher serum creatinine levels predicted poorer outcomes in patients infected with A (H7N9) [6, 7]. In a clinical study of 18 A (H7N9) patients, Lu et al. found that elevated C-creative protein (CRP) predicted patient mortality [8]. A negative correlation has also been observed between the severity of H7N9 infection and the percentage of lymphocytes [4].

Guangzhou is located in the southern portion of Guangdong Province, in the center of the Pearl River Delta. On January 11, the first case of A (H7N9) was identified, and by April 8, 2014, 25 cases had been reported. Fourteen patients (56 %) had died. This death rate was much higher than that of other areas, which ranged from 10 % in Hangzhou city [9] to 39.4 % in Shanghai [10]. This is of particular concern, as Guangzhou is a highly developed city in China with an advanced medical infrastructure, and the HA and NA sequences of the H7N9 viral isolates from Guangzhou patients are identical to those of H7N9 virus strains recently isolated from human patients in other areas (A/Anhui/1/2013, A/Shanghai/1/2013). The present retrospective study on A (H7N9) patients in Guangzhou was conducted to better describe the clinical characteristics of human A (H7N9) patients and evaluate the influential factors associated with the clinical outcomes for the management of high risk patients.

## Methods

### Enrollment of patients

The diagnoses were based on the “Diagnostic and treatment protocol for human infected with avian influenza A (H7N9)” issued by the National Health and Family Planning Commission [11]. Patients with confirmed infection were defined as those with clinical symptoms consistent with acute influenza (fever, coughing, coryza, difficulty in breathing) or with a history of contact with a confirmed or suspected case and a positive laboratory result for the A (H7N9) virus on real-time reverse transcription-PCR. Twenty-five patients were diagnosed with A (H7N9) infection between January 11 and April 10. For comparison, we also studied 16 uninfected patients who were hospitalized during the same period as the A (H7N9)-infected patients. The age, sex and underlying diseases of the 16 controls were matched to those of the A (H7N9)-infected patients.

### Data collection

Field investigations were performed by the staff of the Guangzhou Center for Disease Control immediately after the patients were diagnosed, with face-to-face interviews with the patients, family members, and relevant medical staff. Epidemiological information was collected, including age, sex, underlying disease (including chronic respiratory, gastrointestinal, and cardiovascular diseases, nutritional and metabolic diseases (diabetes, obesity [BMI ≥ 28], and hypoproteinemia)) and important timelines of medical treatment (e.g. time from illness onset to first medical care). The clinical data were extracted retrospectively from the medical records after the patients were discharged from the hospital or died. Clinical data, including routine blood test results (leukocyte, lymphocyte, and neutrophil counts, etc.), liver function (ALT, AST), lactate dehydrogenase (LDH)), coagulation, and renal function (serum creatinine (CRE)) were collected.

### Statistical analysis

Descriptive statistics were used to analyze the epidemiological and clinical characteristics of the A (H7N9) patients. Fisher’s exact test or the Mann-Whitney test was used to compare the differences between fatal and nonfatal cases, between cases that developed into ARDS (‘ARDS cases’) and cases that did not (‘NARDS cases’), and between the controls and the A (H7N9)-infected patients. Based on the normal ranges, the laboratory values were dichotomized into normal or abnormal levels. *P*-values were derived from two-tailed tests and those lower than 0.05 were deemed to be significant. Multivariate analysis was not carried out due to the small sample size. All statistical analyses were performed using SPSS 21.0 (SPSS Inc., Chicago, IL, U.S.).

### Role of sponsors

The sponsors of the study had no role in the study design, data collection, data analysis, data interpretation, or writing of the report. The corresponding authors had full access to all the data in the study and had final responsibility for the decision to submit for publication.

## Results

### Epidemiological characteristics

All 25 patients were hospitalized. The median age was 65 years (range, 4–88), 64 % (16/25) of them were male, and the male:female ratio was approximately 1.8:1. Overall, 84 % (21/25) had one or more underlying diseases. More than half the patients had a chronic cardiovascular disease, and 44 % reported a history of nutritional and metabolic disease. Among the 25 patients, 19 were admitted to an intensive care unit with severe lower respiratory tract disease. Of these 25 patients, 21 required ventilation, and 10 who required invasive ventilation, and 11 who required noninvasive ventilation (using continuous positive airway pressure to provide ventilatory support without an invasive artificial airway). Fifteen patients developed moderate to severe ARDS, 14 patients (56 %) died of ARDS or multiorgan failure. Fisher’s test showed that patients with cardiovascular disease had a higher probability of developing ARDS (*P* = 0.049) (Table 1).

**Table 1:** Epidemiologic and clinical characteristics of 25 patients with laboratory-confirmed influenza A (H7N9) virus infection in Guangzhou

Characteristic	Case	Percent (%)	Death (%)			ARDS (%)		
			Fatal (*n* = 14)	Non-fatal (*n* = 11)	*P*	No (*n* = 10)	Yes (*n* = 15)	*P*
Age (median,range)	65	4–88	71.5 (55–83)	45 (4–88)	0.139	55 (5–79)	65 (59–78	0.317
Sex								
Male	16	64.0	10 (71.4)	6 (54.5)	0.434	5 (50.0)	11 (73.3)	0.397
Female	9	36.0	4 (28.6)	5 (45.5)		5 (50.0)	4 (26.7)	
Type of residence								
Urban	13	52	8 (57.1)	5 (45.5)	0.695	5 (50.0)	8 (53.3)	1.000
Rural	12	48	6 (42.9)	6 (54.5)		5 (50.0)	7 (46.7)	
Required mechanical ventilation	21	84.0						
invasive	10	40.0	6 (42.9)	4 (36.4)	0.040	2 (20.0)	8 (53.3)	0.023
Non-invasive*	11	44.0	8 (57.1)	3 (27.3)		4 (40.0)	7 (46.7)	
Not required	4	16	0 (0.0)	4 (36.4)		4 (40.0)	0 (0.0)	
Underlying diseases (Yes)	21	84.0	12 (85.7)	9 (81.8)	1.000	8 (80.0)	13 (86.7	1.000
Any chronic respiratory disease	3	12.0	2 (14.3)	1 (9.1)	1.000	2 (20.0)	1 (6.7)	0.543
Any chronic gastrointestinal disease	5	20.0	2 (14.3)	3 (27.3)	0.623	3 (30.0)	2 (13.3)	0.358
Any chronic cardiovascular disease	14	56.0	10 (71.4)	4 (36.4)	0.116	3 (30.0)	11 (73.3)	0.049
Any Nutritional and metabolic diseases	11	44.0	7 (50.0)	4 (36.4)	0.689	3 (30.0)	8 (53.3)	0.414

^*^Using continuous positive airway pressure to provide ventilatory support without an invasive artificial airway

### Clinical characteristics

#### Initial symptoms for patients with A (H7N9) infection

**Fig. 1:**
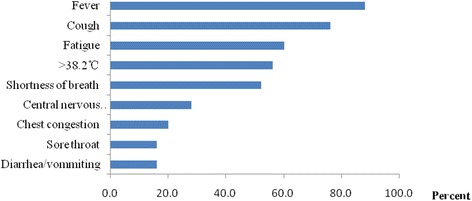
Initial symptoms of 25 patents with confirmed H7N9 virus infection in Guangzhou

Figure 1 showed the initial symptoms of the A (H7N9)-infected patients. Fever was the most common symptoms, with 14 patients complaining of body temperatures over 38.2 °C upon admission. This was followed in frequency by fatigue (62.5 %) and coughing (76 %). Seven (28 %) patients were hospitalized due to symptoms of the central nervous system. The frequency of the initial symptoms did not differ significantly between fatal and nonfatal groups nor between the ARDS and NARDS groups.

#### Timelines for medical care

The median time from onset of illness to first medical care was 1 day (range, 0–7 days). Time from onset to hospitalization was 4 days (range, 0–14 days), and time from onset to confirmation was 7 days (range, 1–14 days). Here, 24 cases received oseltamivir treatment after a median of 5 days (range, 0–11 days) from the onset of symptoms, with 5 patients receiving oseltamivir treatment within 48 h of onset. The 14 patients who died did so at a median of 10 days after onset. According to the Mann-Whitney test, there was no significant difference between the fatal and nonfatal groups with respect to time elapsed from onset of illness to first medical treatment, to hospitalization, to initiation of oseltamivir treatment, or to confirmation of patients. Nevertheless the patients with longer interval from the illness onset to oseltamivir treatment had a higher probability for developing ARDS (*P* = 0.024) (Table 2).

**Table 2:** Medical care Timelines and Clinical Characteresitic of 25 patents with confirmed A (H7N9) virus infection in Guangzhou

Clinical characteristic	Median	Range	Death (Range)			ARDS (Range)		
			Fatal (*n* = 14)	Nonfatal (*n* = 11)	*P*	No (*n* = 10)	Yes (*n* = 15)	*P*
Time from illness onset to first medical care (days)	1	0–7	1.5 (0–6)	1 (0–7)	0.972	0.5 (0–4)	2 (0–7)	0.059
Time from illness onset to hospitalization (days)	4	0–14	4.5 (0–14)	4 (0–7)	0.783	3.5 (0–7)	5 (0–14)	0.109
Time from illness onset to Confirmation (days)	7	1–14	7 (3–14)	7 (1–13)	0.075	6 (1–13)	7 (3–14)	0.485
Time from illness onset to oseltamivir treatment (days)	5	0–11	6 (0–11)	4 (0–8)	0.802	4 (0–7)	6 (0–11)	0.024
Time of sustained viral shedding^a^ (days)	3	1–21	2 (2–11)	3 (1–16)	0.42	3 (2–13)	2 (0–21)	0.199
Time from illness onset to development of ARDS (days)	7	2–14	7.5 (2–14)	7 (6–8)	0.661			
Time from illness onset to development of death (days)	10	2–70						

^a^Patients were followed daily in throat swabs or endotracheal secretions in intubated patients by RT-PCR

#### Laboratory data

As shown in Table 3, the clinical characteristics of the A (H7N9)-infected patients included lymphocytopenia (80 %), elevated CRP (84 %), elevated AST (72 %), and elevated LDH (80 %). More than half the patients had an increased activated partial thromboplastin time (APTT). Only 36 % of patients displayed leukocytopenia, and 20 % displayed thrombocytopenia. The control subjects displayed lymphocytopenia (68.8 %) and neutrophilia (62.5 %). A comparison of the healthy controls and patients with A (H7N9) showed that the A (H7N9) patients were more likely to show leukocytopenia (*P* = 0.024), longer APTT (*P* = 0.002), and elevated AST (*P* = 0.001), LDH(*P* = 0.002), and creatine kinase (CK; *P* = 0.009). Univariate analyses of the patients with fatal and nonfatal infections and of the ARDS and NARDS groups showed that the risk of death was higher in patients with elevated CRP (*P* = 0.026). Patients with elevated CRP (*P* = 0.017), AST (*P* = 0.007), CK (*P* = 0.002), or lymphocytopenia (*P* = 0.005) had a significantly higher probability of developing ARDS.

**Table 3:** Clinical characteristics of 25 patents with confirmed A (H7N9) virus infection in Guangzhou and 16 healthy controls

Clinical characteristic	Health controls *n* = 16	Percent (%)	Case *n* = 25	Percent (%)	*P* ^*^	Death (%)			ARDS (%)		
						Fatal (*n* = 14)	Nonfatal (*n* = 11)	*P*	No (*n* = 10)	Yes (*n* = 15)	*P*
Elevated CRP			21	84		14 (100.0)	7 (63.6)	0.026	6 (60)	15 (100)	0.017
Leukopenia	0	0.0	9	36	0.024	6 (42.9)	3 (27.3)	0.667	3 (30.0)	6 (40.0)	0.691
Lymphocytopenia	11	68.8	20	80	0.472	13 (92.9)	7 (63.6)	0.096	5 (50)	15 (100)	0.005
Thrombocytopenia	0	0.0	5	20	0.374	3 (21.4)	2 (18.2)	1.000	1 (10.0)	4 (26.7)	0.615
Neutropenia	1	6.3	6	24	0.215	4 (28.6)	2 (18.2)	0.664	3 (30.0)	3 (20.0)	0.653
Neutrophilia	10	62.5	7	28	0.029	6 (42.9)	1 (9.1)	0.090	1 (10.0)	6 (40.0 %)	0.179
Elevated TT	0	0.0	7	28	0.114	5 (35.7)	2 (18.2)	0.407	1 (10.0)	6 (40.0)	0.179
Elevated APTT	1	6.3	13	52	0.002	9 (64.3)	4 (36.4)	0.238	4 (40.0)	9 (60.0)	0.428
Elevated ALT	2	12.5	10	40	0.084	6 (42.9)	4 (36.4)	1.000	2 (28.6)	8 (53.3)	0.211
Elevated AST	3	18.8	18	72	0.001	11 (78.6)	7 (63.6)	0.656	4 (40.0)	14 (93.3)	0.007
Elevated LDH	5	31.3	20	80	0.002	12 (85.7)	8 (72.7)	0.623	6 (60.0)	14 (93.3)	0.121
Elevated CK	3	18.8	15	60	0.009	10 (71.4)	5 (45.5)	0.241	2 (20.0)	13 (86.7)	0.002
Elevated CRE	0	0.0	8	32	0.060	6 (42.9)	2 (18.2)	0.234	2 (20.0)	6 (40.0)	0.402

^a^Information was not available for CRP

^*^ *P* value for the comparison between A (H7N9) patients and healthy-controls

#### Lymphocyte subsets

Fifteen (60.0 %) patients displayed reduced percentages of CD3^+^ and CD8^+^ T lymphocytes and 13 (52.0 %) patients displayed reduced percentages of CD4^+^ T lymphocytes. The patients with reduced percentages of CD3^+^(*P = 0.023*) and CD8^+^(*P* = 0.023) T lymphocyte had a significantly higher probability of developing ARDS. However, the levels of lymphocyte subsets did not differ significantly between the patients who died and those who did not (Table 4).

**Table 4:** Lymphocyte subsets of patents with confirmed A (H7N9) virus infection in Guangzhou

Lymphocyte subsets^a^	Case (*n* = 25^a^)	Percent (%)	Death (%)			ARDS (%)		
			Fatal (*n* = 14)	Nonfatal (*n* = 11)	*P*	No (*n* = 10	Yes (*n* = 15)	*P*
Decreased CD3+ T lymphocyte	15	60.0	11 (78.6)	4 (36.4)	0.179	3 (30.0)	12 (80.0)	0.023
Decreased CD4+ T lymphocyte	13	52.0	9 (64.3)	4 (36.4)	0.417	3 (30.0)	10 (66.7)	0.102
Decreased CD8+ T lymphocyte	15	60.0	11 (78.6)	4 (36.4)	0.179	3 (30.0)	12 (80.0)	0.023

^a^The information of 2 patients was missing

**Table 5:** Comparison between influenza A (H7N9) patients in Guangzhou and in the first epidemic wave in other places of China

Characteristic	Guangzhou (*N* = 25)		First wave case	
	Case/Days	Percentage/ Ranges	Case/Days	Percentage/ Ranges
Age^a^				
<65 years	12	48	64	57.7
≥ 65 years	13	52	47	42.3
Underlying disease^a^	21	84	68	61.3
ARDS^a^	20	80	79	71.7
Time from illness onset to first medical care (days)^#^	1	0–4	1	0–3
Time from illness onset to hospitalization (days)^#^	4	1–7	4	3–6
Time from illness onset to oseltamivir treatment (days)^#^	5	3–7	6	5–9
Time from illness onset to development of ARDS (days)^#^	7	6–8	7	5–9

^a^Case and Percentage

^#^Days and Ranges

## Discussion

**Fig. 2:**
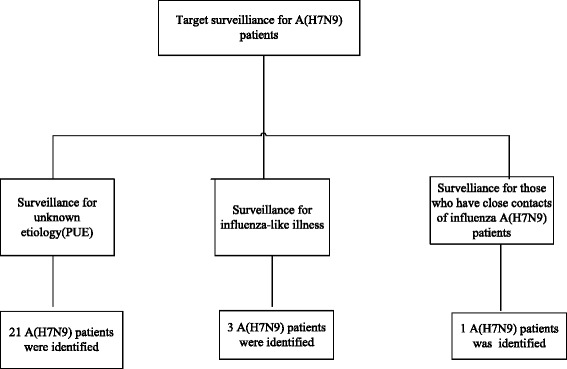
Targeted surveillance for A (H7N9) patients in Guangzhou

This was a retrospective study focusing on the epidemiological and clinical characteristics of patients infected with A (H7N9) in Guangzhou. The clinical outcomes correlated strongly with the timing of oseltamivir treatment and with clinical and biochemical abnormalities. The fatality rate reported here was higher than that in the first epidemic wave in other parts of China (56 % vs. 34 %, respectively) [12]. This may be because 84 % of patients were identified with a surveillance of pneumonia of unknown etiology, which can have a substantially higher risk of mortality than mild cases identified with sentinel influenza-like illness or contract tracing surveillance [13]. The targeted surveillance of A (H7N9) patients is shown in (Fig. 2). An older median age and a high rate of underlying disease might have contributed to the high fatality rate in Guangzhou (Table 3). It is noteworthy that this explanation of the differences in the fatality rate is based on city-collected data. A nationwide database should be established to compare the characteristics of patients from different regions, to explore the possible factors contributing to the discrepancies in fatality rates from A (H7N9) infection.

Our A (H7N9) patients tended to have a high median age and were predominantly males (64 % male vs 36 % females). This pattern is consistent with the results of other studies [14–16], but differs markedly from that of hospitalized A(H5N1) patients. According to a study by Liem et al., the median age of 67 patients with confirmed A(H5N1) viral infection was 25 years (range, 16–42) [17]. The reasons for these different age distributions of patients with A(H5N1) and A (H7N9) are unclear. Several studies have suggested that heterosubtypic cross-immunity could partly explain the different age patterns [18, 19]. A(H5N1) shares the same neuraminidase subtype with A(H1N1) whereas A (H7N9) does not, so elderly people may have been exposed to the A(H1N1) virus during their lives and developed antibodies that cross-react with the A(H5N1), providing some protection against A(H5N1) infection [20]. Elderly patients may become infected with A (H7N9) because they are more likely than younger people to be exposed to live poultry and are more susceptible to the severe form of the disease [21]. The swab samples collected through the surveillance systems for A (H7N9) patients in Guangzhou were primarily from older patients, so it is also possible that younger patients were underreported. Our results show a detectably but insignificantly higher proportion of deaths among elderly patients with A (H7N9) in Guangzhou. The lack of significance might be attributable to the small sample size rather than to a lack of differences between the groups.

A high proportion of patients reported concurrent medical conditions, which is consistent with the results of previous studies. Gao et al. analyzed 111 confirmed cases of A (H7N9) viral infection in patients with comorbidities in parallel with subjects with no underlying medical conditions and found that the presence of a comorbidity was associated with a greater risk of hospitalization with A (H7N9) infection (OR = 3.42, 95 % CI, 1.21–9.70) [21]. Several previous studies have shown that coexisting medical conditions can impair the host’s response to A (H7N9) infection [22, 23]. Moreover, a reduced immune function can allow the survival of less-fit influenza strains, as reported by Nuño et al. [24]. Although a higher proportion of patients with an underlying disease were detected in the group of patients who died than in the nonfatal group, the association between underlying disease and death was not statistically significant. However, patients who had any chronic cardiovascular disease were at higher risk of developing ARDS. Similar patterns have also beenc observed in patients with human influenza infection [25].

According to the clinical data, the initial symptoms of A (H7N9) infection were primary fever, coughing, and fatigue. These findings are consistent with those of other published reports. [9, 10, 26, 27] The atypical symptoms recorded during the early stage of A (H7N9) patients were noteworthy. Two elderly patients were hospitalized due to neurological syndromes. The symptoms of A (H7N9) virus infection were covered up by the neurological syndromes which cause the delay for treatment. Given the high case fatality and features of patients in Guangzhou, it is important to remain on alert for those high risk people. Prior studies have suggested that the time of onset of symptoms may be associated with the fatal clinical outcomes. For example, Shinde reported fever, sore throat, and vomiting to be more common in fatal A(H5N1) cases than in nonfatal cases [28]. In the current case series, the clinical presentation at hospital admission in the fatal, nonfatal, ARDS, and NARDS groups were similar.

The median time interval between the onset of illness and first medical care (first medical visit, hospitalization, or confirmation) is similar to those reported in Shanghai, and the national case report [12, 29]. Neither the duration of illness onset, time of first medical care, nor the median time from onset of illness to confirmation were correlated with clinical outcome. The median time from illness onset to death was 10 days. Several studies have indicated that the progression of influenza A (H7N9) infection was slower than of influenza A(H5N1) diseases [30, 31]. In a recent report, researchers compared the epidemiological features of patients infected with A (H7N9) to those infected with A(H5N1) and found the median time from hospital admission to death to be 12.0 days for patients infected with A (H7N9) and 5.7 days for patients infected with A(H5N1) [32]. The natural history of viral infection, patient characteristics, and differences in clinical management may have contributed to these results. However, a comparison of detailed clinical information between the two groups is still needed.

Oseltamivir treatment was initiated at a median of 5 days after onset of illness (range, 0–11), which was shorter than in the cases reported during the first wave (Table 5) [12]. Early oseltamivir treatment was reported to be effective in reducing the duration of symptoms, the severity of complications, and the fatality rate among patients who were infected with either A(H5N1) virus or pandemic A(H1N1) virus [29, 31]. A randomized placebo-controlled trial found the duration of influenza illness symptoms to be shorter in the oseltamivir group (median,3 days, IQR 1–5 days) than in placebo group (median, 4 days, IQR 1–6 days; *P* = 0.01) [33]. Several studies suggested that oseltamivir treatment, even when started 48 h after onset of disease or later was associated with falling viral load in the respiratory tract in most patents with A (H7N9) infection [21, 34]. The current results also showed that a longer interval between onset of illness to the initiation of oseltamivir testament predicted a higher probability for developing ARDS. For this reason, early treatment of oseltamivir for A (H7N9) patients is here strongly recommended. However, the current results were based on observational study. Well-designed clinical trials are needed to evaluate the efficacy of therapy for patients infected with A (H7N9).

In the present study, ARDS was the primary cause of death, and patients with serious complications had a higher probability of death. Leukocyte counts were generally neither extremely high nor low, but most patients with a poor clinical outcome displayed lymphocytoopenia, which appeared to be a common abnormality among the A (H7N9) patients. Reduced T lymphocytes and their subgroups were observed in 15 patients and the levels of CD3^+^ and CD8^+^ T lymphocytes correlated significantly with the clinical outcome, which is consistent with the results of previous studies. Diao et al. found significantly reduced proportions of CD3^+^ T cells among patients with severe A (H7N9) infection [4]. Chen reported that the proportion of peripheral blood T cells was inversely related to the clinical APACHE II score and the H7N9 viral load [35]. These results suggest that an imbalance in the immune system correlates with the clinical outcomes of A (H7N9) infection and that lymphocytopenia is an early predictor of severe illness.

Most patients showed acute liver injury. Elevated AST was observed in 70 % of the patients and predicted an unfavorable clinical outcome, as reported in previous studies. Liu et al. collected information on 40 patients infected with A (H7N9) and found that elevated AST was significantly closely associated with their prognosis [5]. Hen reported that the AST levels of patients who died from A(H5N1) infection were significantly higher than those of survivors [36]. However, there was no relationship between elevated ALT and clinical outcome. This is because most patients (53.3 %) with normal ALT levels in our study displayed elevated AST. AST is a more sensitive index than ALT in assessing liver injury, and indicated the severe impairment of liver function among the patients with ARDS. Therefore, the negative results do not exclude a relationship between impaired liver function and poor clinical outcome. The laboratory data for humans infected with the A(H1N1) and A(H5N1) viruses shows visibly elevated CK, which predicts a severe, negative clinical outcome. This is consistent with the current results for A (H7N9) patients. In our case series, 60 % of patients showed elevated CK levels, and high CK levels strongly predicted the development of ARDS.

The primary limitation of this study was the small sample size, which restricted the power of the study in identifying significant variables. A patient database that includes cross-regional data should be created. Furthermore, clinical information regarding oseltamivir treatment, such as the treatment schedule, is not given here in detail, and no information on the serum levels of inflammatory cytokines was available in our study because they were not routinely tested in the hospital. The host immunological response plays an important role in disease progression, especially in those who develop severe symptoms. Prospective studies are required to examine the relationship between serum inflammatory cytokine levels and the clinical outcomes of patients with A (H7N9). This case series may have been skewed toward more severe cases because only a small number of patients with mild cases that did not progress to ARDS were included. Therefore, a serological study is required to describe the spectrum of A (H7N9) infection in Guangzhou.

## Conclusions

This study provides a usable estimate of important epidemiological and clinical factors in patients infected with A (H7N9) and some clues to the clinical management of severe cases. The presence of underlying disease and clinical complications predicted poor clinical outcomes and early oseltamivir treatment was associated with a reduced risk of developing ARDS.
